# Identifying Challenges, Enabling Practices, and Reviewing Existing Policies Regarding Digital Equity and Digital Divide Toward Smart and Healthy Cities: Protocol for an Integrative Review

**DOI:** 10.2196/40068

**Published:** 2022-12-08

**Authors:** Tanvir C Turin, Sujoy Subroto, Mohammad M H Raihan, Katharina Koch, Robert Wiles, Erin Ruttan, Monique Nesset, Nashit Chowdhury

**Affiliations:** 1 Department of Family Medicine Cumming School of Medicine University of Calgary Calgary, AB Canada; 2 Department of Community Health Sciences Cumming School of Medicine University of Calgary Calgary, AB Canada; 3 The O'Brien Institute for Public Health Cumming School of Medicine University of Calgary Calgary, AB Canada; 4 Department of Geography University of Calgary Calgary, AB Canada; 5 Department of Sociology University of Calgary Calgary, AB Canada; 6 The School of Public Policy University of Calgary Calgary, AB Canada; 7 Community Strategies The City of Calgary Calgary, AB Canada; 8 Smart Cities, Information Technology The City of Calgary Calgary, AB Canada

**Keywords:** healthy city, smart city, digital equity, digital divide, digital literacy, equity, urban community, inequality, urban area, challenges, barriers, participation, social interaction, structural inequality

## Abstract

**Background:**

Digital equity denotes that all individuals and communities have equitable access to the information technology required to participate in digital life and can fully capitalize on this technology for their individual and community gain and benefits. Recent research highlighted that COVID-19 heightened the existing structural inequities and further exacerbated the technology-related social divide, especially for racialized communities, including new immigrants, refugees, and ethnic minorities. The intersection of challenges associated with racial identity (eg, racial discrimination and cultural differences), socioeconomic marginalization, and age- and gender-related barriers affects their access to health and social services, education, economic activity, and social life owing to digital inequity.

**Objective:**

Our aim is to understand the current state of knowledge on digital equity and the digital divide (which is often considered a complex social-political challenge) among racialized communities in urban cities of high-income countries and how they impact the social interactions, economic activities, and mental well-being of racialized city dwellers.

**Methods:**

We will conduct an integrative review adapting the Whittemore and Knafl methodology to summarize past empirical or theoretical literature describing digital equity issues pertaining to urban racialized communities. The context will be limited to studies on multicultural cities in high-income countries (eg, Calgary, Alberta) in the last 10 years. We will use a comprehensive search of 8 major databases across multiple disciplines and gray literature (eg, Google Scholar), using appropriate search terms related to digital “in/equity” and “divide.” A 2-stage screening will be conducted, including single citation tracking and a hand search of reference lists. Results will be synthesized using thematic analysis guidelines.

**Results:**

As of August 25, 2022, we have completed a systematic search of 8 major academic databases from multiple disciplines, gray literature, and citation or hand searching. After duplicate removal, we identified 8647 articles from all sources. Two independent reviewers are expected to complete the 2-step screening (title, abstract, and full-text screening) using Covidence followed by data extraction and analysis in 4 months (by December 2022). Data will be extracted regarding digital equity–related initiatives, programs, activities, research findings, issues, barriers, policies, recommendations, etc. Thematic analysis will reveal how barriers and facilitators of digital equity affect or benefit racialized population groups and what social, material, and systemic issues need to be addressed to establish digital equity for racialized communities in the context of a multicultural city.

**Conclusions:**

This project will inform public policy about digital inequity alongside conventional systemic inequities (eg, education and income levels); promote digital equity by exploring and examining the pattern, extent, and determinants and barriers of digital inequity across sociodemographic variables and groups; and analyze its interconnectedness with spatial dimensions and variations of the urban sphere (geographic differences).

**International Registered Report Identifier (IRRID):**

DERR1-10.2196/40068

## Introduction

### Background

Access to digital technologies and ensuring digital equity have gained traction in recent policy debates and become priority concerns for transforming smart cities across the world. According to the National Digital Inclusion Alliance, digital equity is defined as “a condition in which all individuals and communities have the information technology capacity needed for full participation in our society, democracy, and economy” [1]. When the state fails to ensure the capacity of accessing and using information and communications technology and services among different segments of its people, it is denoted as digital inequity or digital divide [2]. Access to critical services, jobs, lifelong learning, and civic and cultural involvement depend on digital equity [1]. This notion has gained further momentum during the COVID-19 pandemic and emerged as a dominant agenda in urban planning [3]. However, despite continued efforts to bridge the digital divide, numerous studies have reported issues of growing digital inequity concerning access to the internet, software, and hardware; level of digital literacy (the ability and skills to use); and adoption of digital technology [4-8]. Nevertheless, this underlying phenomenon of the digital divide is not an isolated thing but is, in fact, embedded in pre-existing structural and systemic inequities [6], often resulting from socioeconomic marginalization and socio-spatial disparities. Therefore, critical calls are increasing for a more careful analysis of the intersectionality of digital inequity with a special focus on the interplay between varying sociodemographic backgrounds or factors and urban socio-spatial factors [6].

Pandemic-induced restrictions and subsequent lockdowns, which had already placed disproportionate burdens on marginalized groups, have diminished (in-person) social interactions and resulted in increased dependency on digital technologies. Previous research highlighted that the COVID-19 pandemic has heightened the existing structural inequities [9] and further exacerbated the technology-related social divide, especially for older adults [10], the economically marginalized, and members of racialized communities (ie, immigrants, refugees, and ethnic minorities) [8,11], by limiting their access to health services [12], economic activity, and social life. Moreover, this crisis has also exposed the multifaceted nature of digital inequities, which are compounded by the ongoing equity challenges, and how they disproportionately impact those vulnerable groups who are already affected by socio-spatial inequities [13]. Emerging research on the pandemic has demonstrated that digital equity is not only a social determinant of health [12] but also a precondition for gaining access to economic activity, social life or sphere, and other urban services. Therefore, given the complexity, multidimensionality, and severity of the crisis for disadvantaged groups resulting from digital inequities, scholars and practitioners have emphasized developing robust mitigation and adaptation strategies by considering the broader socioeconomic [11] and socio-spatial context [13] of urban areas. Since access to digital technology has become fundamental to everyday life, if equity is not ensured, it may reinforce systemic inequity for digitally disadvantaged groups, who may fall behind during the postpandemic recovery phase.

Many studies reported that access to the internet for racialized communities is much lower than the national average [14,15]. Ethnic minorities were found to be significantly more worried (40%-53%) regarding the ability to pay for the internet than their counterparts (29%), according to a report on the digital divide in Toronto, Ontario [16]. Some members of this category are also at risk of the digital divide because of a lack of content accessibility [17], in addition to the barriers to access to devices and subscription vulnerabilities [18]. The capacity and ability of those in racial and ethnic minorities to navigate the digital sphere and space are constrained, which may shape or limit their ability to engage in a variety of complex web-based activities including accessing health [18,19] and social support services [20]. Immigrants and refugees are made up of varied groups with a range of skills and socioeconomic circumstances [21]. For example, economic migrants tend to be highly educated and have digital-literacy skills, whereas family migrants or refugees may have low digital-literacy skills [21]. However, regardless of the subtypes, immigrants and refugees usually undergo resettlement challenges including language, employment, and financial barriers, which may affect the accessibility and affordability of digital devices and services [22].

Previous research has mainly focused on digital inequity in limited-income countries when considering the global context [2,23]. In the context of Canada, most studies specifically have explored the rural-urban divide [15,24]. Previous literature reviews related to digital equities in racialized communities generally focused on motivation for internet adoption and information practices [25,26], the eHealth literacy aspect [27,28], older immigrants [29,30], and social media use [31]. What remains understudied is how systemic inequities and various social determinants affect the racialized population in multicultural urban centers, where people from diverse backgrounds and socioeconomic capacities thrive under the same jurisdiction, governing bodies, and supposedly same facilities of internet and digital services, yet live on the extreme ends of the digital equity spectrum [32]. There is a need for understanding how the intersection of various systemic inequities (racism, discrimination, ableism, etc) and characteristics of racialized communities (eg, culture and language) lead to and exacerbate existing digital inequities among racialized communities [33]. This study, therefore, aims to understand this complex issue by drawing on previously published studies and inform public policy on treating digital inequity by illustrating the interconnectedness of digital equity with systemic inequities and its spatial variations in the urban sphere.

### Study Objectives

We intend to capture the current understanding of digital equity through an integrative review of academic and gray literature to achieve the following two specific objectives:

- Objective 1: we plan to explore the current level of research regarding digital inequity and synthesize the knowledge of the barriers and facilitators and potential outcomes of digital inequity. This understanding will help us determine and undertake the next steps in working on this important but overlooked issue.
- Objective 2: we plan to identify the reported initiatives for overcoming digital inequity in racialized communities. Having this information will allow different levels of stakeholders in this area to access the preliminary knowledge to undertake solution-oriented research and program initiatives.

## Methods

### A Community-Engaged Research Approach

As a part of a community-engaged program of research, we strive to engage with various communities through knowledge cocreation, knowledge comobilization, and equitable partnership strategies where the partners have decision-making capacities across the steps of the research process [34,35]. Community members, community champions, citizen researchers, nonprofit organizations, and policy makers such as municipalities, local government bodies, and others are involved in our research program at various levels of capacity [36]. Through our outreach activities, we have the opportunity to engage with the City of Calgary, which identified a research need regarding digital inequity, to explore why and how digital equity affects racialized communities in Calgary. Therefore, together with the city team, we developed the study protocol, which seeks to synthesize knowledge from existing research, policies, programs, and initiatives on this issue in the urban context in high-income countries. Textbox 1 presents the guiding questions of this research. The knowledge obtained through this study will allow us to understand the current extent of the research regarding digital equity and will inform policy makers and community partners to develop strategies to effectively address existing inequity.

**Guiding questions.**

1. To map the publications about digital inequity and the digital divide focusing on racialized communities
2. To summarize the existing policy, strategy, interventions, regulatory frameworks, and recommendations against digital inequity in racialized communities
3. To identify the key determinants of digital inequity in racialized communities
4. To explore the key constructs and dynamicity of the digital divide in racialized communities
5. To inspect the partnership approaches across actors and stakeholders used in digital equity initiatives and programs

### Systematic Integrative Review

#### Overview

We will be conducting a systematic integrative review using Whittemore and Knafl’s [37] methodological approach. Integrative reviews gather comprehensive knowledge from both empirical and theoretical literature and allow a better understanding of a particular issue [38]. This review approach does not place restrictions on a certain methodology, thus allowing evidence on a particular topic to be illustrated from a broader perspective, which helps in developing theories and practices [37]. To ensure rigor, we will adapt the guidelines from the PRISMA-P (Preferred Reporting Items for Systematic Review and Meta-Analysis Protocols) for this integrative review to enhance methodological and reporting quality [39] (Multimedia Appendix 1).

#### Identifying the Problem

Based on previous studies and our community engagement activities, we observed inequities across various population groups and areas in terms of accessibility, availability, and affordability of internet connection; internet-enabled devices; digital literacy and skills; and useful materials, resources, and outcomes [15,40]. Through our discussions with the City of Calgary and community partners, we coidentified the questions that will guide our study (Textbox 1).

#### Literature Search

Using the PCC (Population, Concept, Context) framework [6], we developed the following inclusion and exclusion criteria.

##### Population

For this integrative review, we will include studies conducted among the urban racialized population including immigrants, refugees, and ethnic minorities. Our focus is on various groups of the racialized populations in urban contexts, as in comparison to rural dwellers, who are more likely to be able to avail themselves of the infrastructure necessary to access high-speed internet if they were not affected by various systemic inequities [41]. We define high-speed internet as 50 Mbps download/10 Mbps upload in accordance with the Canadian Radio-television and Telecommunications Commission’s service objective [42].

##### Concept

Our search parameters will include any type of research project, as well as pilot, temporary, and experimental studies addressing digital equity and sustainable and failed initiatives, programs, or activities aimed at establishing digital equity. In this review, we will interpret digital equity, equality, or inclusion similarly, a concept we define as having access to and the capacity to utilize information technology with positive outcomes by all individuals and communities [1]. Any disruption to this concept, such as certain individuals or communities being unable to access the internet or utilize available digital technology, is denoted as digital inequity, inequality, divide, exclusion, or gap in this study. All types of studies, including, but not limited to, exploring barriers, facilitators, outcomes, policies, reforms, and so on, will be considered in this review.

##### Context

Based on discussions with our partners, we want to focus on studies conducted in multicultural and high-income cities similar to the City of Calgary, which is a cosmopolitan city of approximately 1.4 million people in Alberta, Canada. Therefore, we will include studies in urban areas of high-income countries. High-income countries will be selected using the United Nations’ list of countries with high-income economies [43]. We will include studies that address structural, technological, legal, business, and other aspects of internet access and quality, increased accessibility, availability, and affordability of internet-enabled devices, and improvement of digital skills and literacy that connect their findings or discuss them in relation to digital equity. We will include studies about digital equity in any context (eg, digital equity in health care access, law and order, social support, employment and economy-related aspects, etc).

##### Search Strategy

We will keep our inclusion criteria broad in terms of types of study design. We plan to capture the maximum possible work undertaken on this topic, so we will include original and review studies, qualitative, quantitative, and mixed method studies, theses and dissertations, editorials, commentaries, and case studies. We will not, however, include books or book chapters in this review. We will only include English-language studies and studies published since January 2010.

We have developed a search strategy in consultation with a librarian and following the evidence-based Peer Review of Electronic Search Strategies (PRESS) guideline [7]. The strategy is designed to capture both peer-reviewed journal-published articles and gray literature from sources from multiple disciplines, including social sciences and humanities, computer science and technology, and health sciences. A list of keywords, index terms, and search algorithms is presented in Textbox 2. We have also provided a detailed search strategy for 1 database—MEDLINE (Multimedia Appendix 2). It is important to note that different databases have different search mechanisms, and we will adapt search strategies accordingly. For example, the search keywords and combinations used in MEDLINE will need to be modified for Scopus to yield optimal search outcomes. To further ensure our search is extensive, we will review the reference lists of the initially selected studies to elicit additional articles we may have missed during our initial search. The academic and gray literature databases we will search for this review are provided in Textbox 3. As gray literature will contain a wide variety of non–peer-reviewed publications, we will apply the AACODS (authority, accuracy, coverage, objectivity, date, and significance) checklist to ensure the credibility and validity of the information from each data source [44].

**Search terms and search strategy.**

**Keywords for digital (in)equity**

(Digital* OR “digital literacy” OR “information technology” OR “digital technology” OR technology OR internet OR “information and communications technology” OR ICT OR computer OR mobile OR phone OR smartphone OR “smart devices” OR cyber OR web OR “data literacy” OR “information literacy”) AND

**Keywords for** **high-income countries**

(“OECD countr*” OR “developed countr*” OR “Western countr*” OR “Organisation for Economic Co-operation and Development” OR “developed nation*” OR “advanced countr*” OR “advanced nation*” OR “industrialized nation*” OR “industrialized countr*” OR “high-income countr*” OR “first world count*” OR “MEDC countr*” OR “More economically developed countr*”) AND

**Keywords for (in)equity**

(Equity OR inequity OR divide OR inclusion OR exclusion OR gap OR inequality OR apartheid OR equality OR disadvantage* OR inconvenien* OR access* OR unfair OR fair OR justice OR injustice OR discrimination OR bias OR unjust OR need* OR barrier* OR obstacle* OR limitation* OR deficit OR shortage OR inadequate OR poverty OR scarcity OR insufficient OR scant)

**Academic and gray literature databases.**

**Academic articles**

- Web of Science
- Scopus
- Academic Search Complete
- Canadian Research Index
- MEDLINE
- SocINDEX with Full Text
- Communication & Mass Media Complete
- IEEE Xplore digital library: Standards

**Gray literature**

- Google Scholar
- ProQuest (theses and dissertations)
- OAISter (WorldCat)
- National Digital Inclusion Alliance
- Canadian Radio-television and Telecommunications Commission

#### Evaluating Data

**Figure 1:**
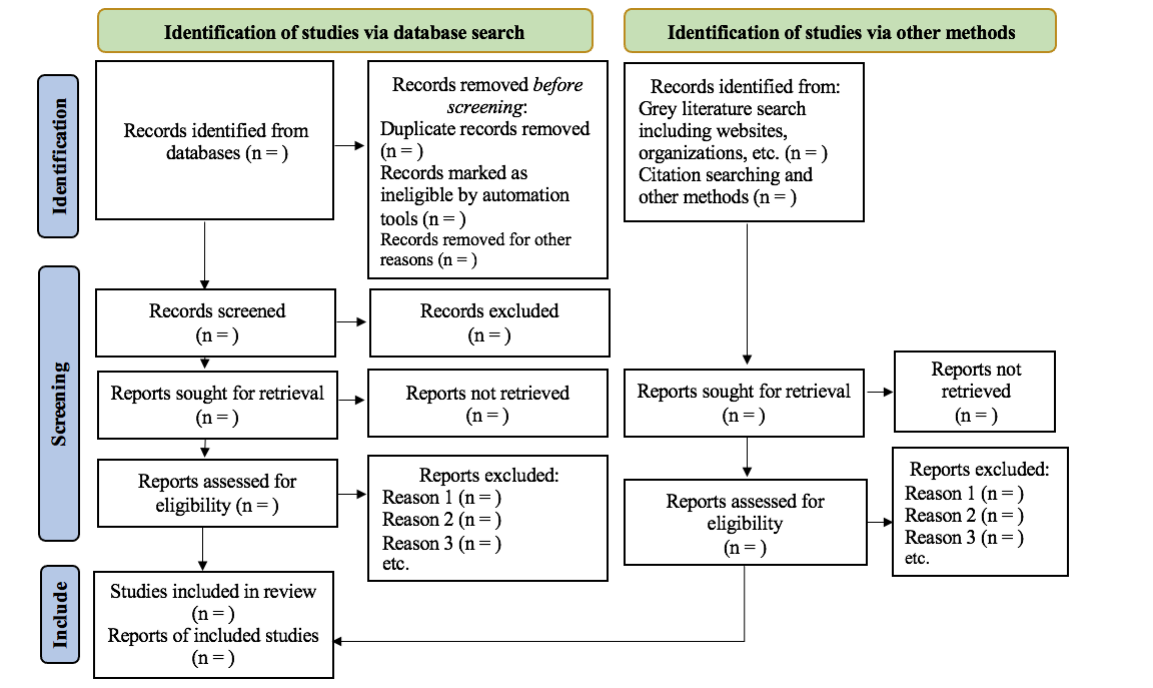
Flow diagram of the search, screening, and selection process for the review.

All identified records following the search will be uploaded into Covidence, a systematic review tool, and duplicates will be removed. Initially, the title and abstract will be screened by 2 independent reviewers to identify potential studies for full-text review according to the inclusion criteria stated above. Potentially eligible articles during title/abstract screening will be thoroughly screened by the same reviewers for final eligibility. Articles fulfilling all inclusion criteria will be selected for this review. The agreement between the 2 reviewers is expected to be 80% or greater. Any conflicts between the 2 reviewers will be resolved by a discussion including a third reviewer. Each step of the study-selection process will be documented and reported using an adapted version of the PRISMA-P flow diagram (Figure 1) [5].

#### Data Analysis

The data from the eligible articles will be charted and collated by 2 independent reviewers. We have developed a preliminary data extraction instrument (Textbox 4). Nevertheless, as the reviewers go through the studies, they may find new themes and interesting information to extract, and those will be added to the extraction tool. The study characteristics (author, year of publication, methodology and methods, location and context of the studies, objectives and research questions, and population demographics) will be extracted from each study. In addition, we will extract information related to digital equity, such as the barriers, facilitators, outcomes, details of a project and how they were conducted, key findings from the research, recommendations, and future research directions. In case of any disagreements between the reviewers, a third reviewer will mediate to arrive at a consensus. If there is any missing data in the eligible articles, the authors of those papers will be contacted.

We will go through the following phases during the analysis based on the thematic analysis framework by Braun and Clarke [45]:

- Data familiarization: includes an iterative reading of the articles and highlighting interesting points related to the research questions.
- Generating initial code: double-checking the initial highlighted points and identifying new codes or modifying them as initial codes if they represent a specific idea relevant to the research questions.
- Searching for themes across the data: compare and contrast to identify themes and subthemes from the coded data.
- Reviewing themes: through discussion among the research team, the themes will be reviewed to ensure they align with the different perspectives of the team members, including the researchers, city, and community stakeholders of this topic.
- Producing the report: a scholarly report will be produced for peer-reviewed publications.

**Data extraction scheme.**

**Citation details**

- Title
- Authors
- Publication date
- Country of publication
- Journal
- Type of publication

**Study demographics**

- Participant demographics
- Sample size
- Population subgroups
- City or cities where the project was undertaken
- Stakeholders involved (researchers, policy makers, etc)
- Constructs of the digital divide

**Digital equity characteristics**

- Primary aim of the study or initiative
- Aspects of digital equity (ie, access, availability, skills, etc)
- Explorative or solution-oriented
- Study focus level (ie, community, city administration, etc)
- Duration or frequency
- Sustained, temporary, pilot, or failed

**Initiative/program details**

- Description
- Justification
- Recruitment
- Challenges described
- Facilitators described
- Key steps or process description

**Study findings**

- Outcomes
- Recommendations
- Future research directions
- Future implementation direction
- Applicability
- Limitations, gaps, or concerns

#### Presenting the Results

Following step 4 above, the extracted data will be iteratively compared, scrutinized, and discussed between the research team to generate key themes and subthemes. In the manuscript, the extracted data will be presented in tabular or diagrammatic form, while a summary and lessons learned will be presented in a narrative format.

The results will be organized based on the key themes and subthemes, and a summary will be generated for meaningful interpretation. The knowledge gained from the studies will be interpreted in light of our research questions and will be presented so that potential knowledge users, such as the City of Calgary and other stakeholders, can utilize it. Any research gaps will also be pointed out to provide future research directions.

## Results

As of August 25, 2022, we have searched 8 academic databases from multiple disciplines (Web of Science, Scopus, Academic Search Complete, Canadian Research Index, MEDLINE, SocINDEX with Full Text, Communication & Mass Media Complete, and IEEE Xplore digital library: Standards). We identified 9776 articles from the search results initially and uploaded them into Covidence. Covidence removed 1312 duplicates, resulting in 8464 articles to be screened. In addition, a gray literature search including Google Scholar, ProQuest (Theses and Dissertations), OAISter (WorldCat), Google, Bing, Yahoo!, and several organizational websites (National Digital Inclusion Alliance and Canadian Radio-television and Telecommunications Commission) were searched. Initially, we identified 178 articles from the search results, and 5 more articles have been sourced by our partner, the City of Calgary’s connection in other municipalities and provinces. Two independent reviewers will complete the 2-step screening (title, abstract, and full-text screening) of a total of 8647 articles followed by data extraction and analysis in 4 months (expected by December 2022).

## Discussion

### Anticipated Outcomes

We intend to identify and summarize key findings from existing digital equity–related initiatives, programs, activities, research findings, issues, barriers, policies, recommendations, etc from the peer-reviewed literature. This will give us an understanding of the landscape of research and initiatives that have been systematically reported. We expect to learn what barriers and facilitators of digital equity exist, which population groups are being affected the most and why, and what social, material, and political issues need to be addressed to establish equity in the context of a high-income and multicultural city. We will learn the findings and recommendations from research projects on digital equity and descriptions of which approaches may or may not work and why and the thoughts and behaviors of community members and private, nonprofit, and government stakeholders. From the gray literature, that is, non–peer-reviewed organization reports and reflections on digital equity–related programs and policies, we will learn about practical experiences from the implementation perspective. The integrative review will also allow us to understand the available and necessary resources in respect of digital equity and how to acquire more resources and apply them in an appropriate way.

This study has a narrow focus on digital equity in racialized communities in the urban areas of high-income countries. In an earlier period, ensuring internet connection and accessibility of internet-enabled devices were the key issues against digital equity in urban areas, which is often termed in the literature as the first level of the digital divide [46,47]. However, having an internet connection and internet-enabled devices accessible and available has shifted the focus of concern toward the second and third levels of the digital divide. The second level refers to the improvement of digital literacy of the urban population, while the third level focuses on enabling them to gain the maximum output (eg, gaining employment or health services using the internet) [48]. Improving individual digital skills or literacy, including using the internet and understanding and ensuring one’s digital privacy, contributes to a gain in digital capital that may contribute to human, economic, and social capital [49]. However, despite having the same level of digital skills, the same 2 people may not benefit at the same level. For example, one may want to learn more about a certain physical condition, but the information on the website is only available in medical terms and not in plain language. Therefore, with the same digital skills, a medically savvy person would gain more from the internet than one who is less so. Further, information may be available in one language but not in another, which also creates a discrepancy between the outcome levels for different users. Digital equity also improves trust in web-based activities and persons on the other side of the digital communication, thus increasing social interaction and harmony [49].

### Strengths and Limitations

A key strength of this study protocol is its comprehensiveness in including both a traditional academic literature review and an internet scan. It also ensures rigor by following methodological frameworks for both types of activities. The research team is also a profound strength of this study, as partners represent several stakeholders, including the city, community, and multidisciplinary researchers. We have ensured that each of the research team members provides input in developing the search, screening, and analysis strategies and that all perspectives are addressed. In the same fashion, we also have generated a data extraction tool that ensures we extract the maximum relevant data and are able to generate meaningful themes and subthemes.

We also acknowledge certain limitations in this protocol. Digital equity is a very vague and multisectoral topic and can be viewed from numerous perspectives and contexts. For example, digital equity may mean one thing in medicine while certain issues may not apply in education. While we will attempt to capture studies from all disciplines, it might prove overwhelming to attempt to gather all elements of the topic most salient to every discipline and sector in this proposed study. In addition, while there may be certain similarities between cities, each city is unique and has its own strengths and limitations in relation to the topic. While it is crucial to know what activities have been undertaken in cities similar to Calgary to promote digital equity among racialized communities, their approaches and implementation may not be applicable to other cities.

### Community-University Partnership and Dissemination Plan

We are taking a community-engaged research approach in this study [50] following the principles of integrated knowledge translation [51,52] where the research partner is involved in each step of the research process from research design to dissemination. Such involvement of the partner accelerates the research uptake and implementation. As our research questions originated from the knowledge user, our partner organization, the City of Calgary, and as they will be involved in each following step of the study, the knowledge generated here will be directly transferred to the actively involved knowledge users. In addition, we will create a report or policy brief for the City of Calgary stakeholders, which will be distributed by the partners. In addition to a peer-reviewed manuscript conveying the findings of this study to the academics, we will also create an infographic in plain language and a video doodle summarizing the findings in lay terms. We will disseminate these through our social and ethnic media networks to reach the extended group of stakeholders and the racialized communities in Calgary and beyond. The City of Calgary’s digital equity team also has established a cross-sectorial Digital Equity Advisory Panel, and the panel members will be important knowledge users for this review report as well as important knowledge mobilizers of this review’s findings.

### Conclusions

Digital equity is complex to achieve, as it intersects with a variety of systemic inequities. Learning from previous studies and other high-income cities through an integrative review and internet scan will provide valuable insights into future research, development, and policy directions. The urban population is generally extremely diverse, and each population group within an urban area may have unique advantages and disadvantages in terms of digital equity. Being informed about those unique aspects will help develop workable and acceptable strategies to improve digital equity for all.

## Appendix

Multimedia Appendix 1 PRISMA (Preferred Reporting Items for Systematic Reviews and Meta-Analyses) checklist adapted for a systematic integrative review.

Multimedia Appendix 2 Search string (MEDLINE).

## Abbreviations

AACODS: authority, accuracy, coverage, objectivity, date, and significance
PCC: Population, Concept, Context
PRESS: Peer Review of Electronic Search Strategies
PRISMA-P: Preferred Reporting Items for Systematic Review and Meta-Analysis Protocols
