# Supplementary material for: Identifying Challenges, Enabling Practices, and Reviewing Existing Policies Regarding Digital Equity and Digital Divide Toward Smart and Healthy Cities: Protocol for an Integrative Review
Source: JMIR Res Protoc. 2022 Dec 8;11(12):e40068. doi: 10.2196/40068 (PMC9782333; doi:10.2196/40068)
Supplement: Multimedia Appendix 2 [file resprot_v11i12e40068_app2.docx]

Appendix 2. Search string (MEDLINE).

| **Searches** | **Results** | **Type** |
| --- | --- | --- |
| 1 | digital*.mp. or exp Digital Technology/ | 184115 |
| 2 | exp Computer Literacy/ or digital literacy.mp. | 2033 |
| 3 | information technology.mp. or exp Information Technology/ | 14363 |
| 4 | digital technology.mp. or exp Digital Technology/ | 2258 |
| 5 | exp Technology/ or technolog*.mp. | 1005370 |
| 6 | internet.mp. or exp Internet/ | 127810 |
| 7 | (information and communications technology).mp. [mp=title, abstract, original title, name of substance word, subject heading word, floating sub-heading word, keyword heading word, organism supplementary concept word, protocol supplementary concept word, rare disease supplementary concept word, unique identifier, synonyms] | 459 |
| 8 | ICT.mp. | 6632 |
| 9 | cyber.mp. | 2273 |
| 10 | web.mp. | 148777 |
| 11 | data literacy.mp. | 24 |
| 12 | information literacy.mp. or exp Information Literacy/ | 8357 |
| 13 | equity.mp. | 23393 |
| 14 | inequity.mp. | 4828 |
| 15 | divide.mp. | 19685 |
| 16 | inclusion.mp. | 298082 |
| 17 | exclusion.mp. | 117003 |
| 18 | gap.mp. | 176858 |
| 19 | inequality.mp. | 22511 |
| 20 | equality.mp. | 9975 |
| 21 | access*.mp. | 657873 |
| 22 | unfair.mp. | 2602 |
| 23 | fair.mp. | 43798 |
| 24 | justice.mp. | 30951 |
| 25 | injustice.mp. | 1987 |
| 26 | discrimination.mp. | 161145 |
| 27 | unjust.mp. | 774 |
| 28 | need*.mp. | 2171322 |
| 29 | barrier*.mp. | 363587 |
| 30 | exp Poverty/ or poverty.mp. | 66651 |
| 31 | OECD countries.mp. or exp "Organisation for Economic Co-Operation and Development"/ or exp Developed Countries/ | 22206 |
| 32 | Western countries.mp. | 18071 |
| 33 | developed nations.mp. | 2059 |
| 34 | advanced countries.mp. | 451 |
| 35 | industrialized countries.mp. | 7622 |
| 36 | high-income countries.mp. | 9185 |
| 37 | first-world countries.mp. | 78 |
| 38 | MEDC countries.mp. | 0 |
| 39 | More economically developed countries.mp. | 34 |
| 40 | 31 or 32 or 33 or 34 or 35 or 36 or 37 or 38 or 39 | 57762 |
| 41 | 13 or 14 or 15 or 16 or 17 or 18 or 19 or 20 or 21 or 22 or 23 or 24 or 25 or 26 or 27 or 28 or 29 or 30 | 3687513 |
| 42 | 1 or 2 or 3 or 4 or 5 or 6 or 7 or 8 or 9 or 10 or 11 or 12 | 1382832 |
| 43 | 40 and 41 and 42 | 2002 |
